# Characterization of metabolic reprogramming by metabolomics in the oncocytic thyroid cancer cell line XTC.UC1

**DOI:** 10.1038/s41598-023-27461-2

**Published:** 2023-01-04

**Authors:** Tomomi Kurashige, Mika Shimamura, Koichiro Hamada, Michiko Matsuse, Norisato Mitsutake, Yuji Nagayama

**Affiliations:** 1 Department of Molecular Medicine, Atomic Bomb Disease Institute, Nagasaki University Graduate School of Biomedical Sciences, 1-12-4 Sakamoto, Nagasaki, 852-8523 Japan; 2 Department of Community Medicine, Nagasaki University Graduate School of Biomedical Sciences, Nagasaki, 852-8523 Japan; 3 Department of Radiation Medical Sciences, Atomic Bomb Disease Institute, Nagasaki University Graduate School of Biomedical Sciences, Nagasaki, 852-8523 Japan

**Keywords:** Cancer, Cancer metabolism

## Abstract

Oncocytic thyroid cancer is characterized by the aberrant accumulation of abnormal mitochondria in the cytoplasm and a defect in oxidative phosphorylation. We performed metabolomics analysis to compare metabolic reprogramming among the oncocytic and non-oncocytic thyroid cancer cell lines XTC.UC1 and TPC1, respectively, and a normal thyroid cell line Nthy-ori 3-1. We found that although XTC.UC1 cells exhibit higher glucose uptake than TPC1 cells, the glycolytic intermediates are not only utilized to generate end-products of glycolysis, but also diverted to branching pathways such as lipid metabolism and the serine synthesis pathway. Glutamine is preferentially used to produce glutathione to reduce oxidative stress in XTC.UC1 cells, rather than to generate α-ketoglutarate for anaplerotic flux into the TCA cycle. Thus, growth, survival and redox homeostasis of XTC.UC1 cells rely more on both glucose and glutamine than do TPC1 cells. Furthermore, XTC.UC1 cells contained higher amounts of intracellular amino acids which is due to higher expression of the amino acid transporter ASCT2 and enhanced autophagy, thus providing the building blocks for macromolecules and energy production. These metabolic alterations are required for oncocytic cancer cells to compensate their defective mitochondrial function and to alleviate excess oxidative stress.

## Introduction

Thyroid oncocytic carcinoma, also known as Hürthle cell carcinoma, has a unique phenotype that is characterized by its enlarged oxyphilic cytoplasm where mitochondria are aberrantly accumulated^1,2^. In the most but not all of these cancers, mitochondria are dysfunctional due to missense or less frequently disruptive mutations in the mitochondrial DNA (mtDNA) coding for complex I and/or III of the electron transport chain (ETC), which is essential for oxidative phosphorylation (OXPHOS)^3–6^, although the mere presence of such mtDNA mutations does not necessarily mean tumors that the tumors are oncocytic^7^. In addition to a compensatory increase in mitochondrial biogenesis^8–10^, we have recently demonstrated that the oncocytic phenotype (that is, the accumulation of mitochondria) is also caused by defective expression of mitochondria-eating protein (MIEAP)^11^, an indispensable molecule for non-canonical mitophagy (see^12^ for a review). Thus, this type of tumor must totally rely on glycolysis for energy production by shifting from OXPHOS to aerobic glycolysis (called the Warburg effect)^13^. It has been documented that metabolic reprogramming like this is a hallmark of cancer^14^. Indeed it has previously been shown that glucose in the culture medium is indispensable for in vitro growth/survival and ATP production of the oncocytic thyroid cancer cell line XTC.UC1^15,16^. XTC.UC1 cells have also been shown to exhibit a lower basal oxygen consumption rate (OCR) and a higher basal extracellular acidification rate (ECAR)^17,18^. Furthermore, higher expression of glycolysis-related proteins, glucose transporter 1, hexokinase II, carbonic anhydrase IX and monocarboxylate transporter 4, has been demonstrated in oncocytic thyroid cancer tissues compared to non-oncocytic cancer tissues^19^. Although energy production by glycolysis is inefficient from an energetic perspective, it fulfills the biomass requirements for rapid cell proliferation.

However, there have been contradictory data reported; for example, the expression levels of lactate dehydrogenase (LDH) have been shown to be lower in oncocytic thyroid tumors than non-oncocytic tumors^10^. Similar data have also been reported in renal and pituitary oncocytomas, compared to normal kidney tissues and non-oncocytic pituitary adenomas, respectively, showing diminished glycolysis with low glucose 6-phosphate, low to normal lactate and low LDH levels^20,21^. Normalization of mitochondrial complex I function by gene transfer in osteosarcoma cells with impaired complex I function has been shown to increase LDH expression, indicating that a functional complex I is essential for the induction of glycolysis^22^.

These controversial data prompted us to perform metabolomics analyses and compare the metabolic statuses of the oncocytic thyroid cancer cell line XTC.UC1, the non-oncocytic papillary cancer cell line TPC1, and the immortalized normal thyroid cell line Nthy-ori 3-1; the latter two are widely used to compare metabolic states with XTC.UC1 cells^15,18^. Metabolomics is an emerging omics technology that is useful for systematically quantifying metabolites in biological samples. We also generated a mtDNA-defective TPC1 cell line (ρ0 cells) to see if the phenotypic differences between XTC.UC1 and TPC1 cells are due to differences in mitochondrial function.

## Results

### Seahorse analysis

**Figure 1:**
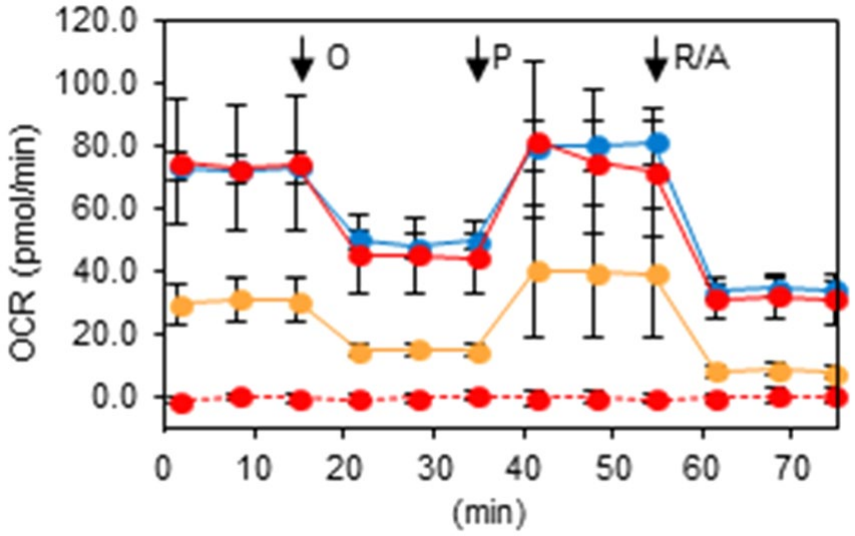
Mitochondrial oxygen consumption rates in Nthy-ori 3-1, XTC.UC1, TPC1 and TPC1 ρ0 cells. The oxygen consumption rates were measured at the basal levels and after 2 μM oligomycin (O), 0.25 μM phenylhydrazone (P) and 0.5 μM rotenone/antimycin (R/A) treatments as described in the “Materials and methods”. Blue, Nthy-ori 3-1; yellow, XTC.UC1; red with solid lines, TPC1; red with broken lines, TPC1 ρ0 cells. Data are presented as means ± SE.

First, we used a Seahorse XF-96 extracellular flux analyzer to compare OCR among XTC.UC1, TPC1, TPC1 ρ0 and Nthy-ori 3-1 cells (Fig. 1). TPC1 and Nthy-ori 3-1 cells exhibited comparably normal patterns of basal and maximal OCR in response to treatment with mitochondrial complex inhibitors, while TPC1 ρ0 cells showed extremely low basal OCR and no response to treatments, confirming normal and completely abolished mitochondrial function in the parental TPC1 and TPC1 ρ0 cells, respectively. XTC.UC1 cells exhibited an intermediate pattern, that is, lower basal OCR and partial response to mitochondrial inhibitors, data consistent with Addie et al.^23^, indicating the ETC in XTC.UC1 cells being partially impaired. In contrast, another group has reported a slightly different data on mitochondrial function in XTC.UC1 cells, indicating almost complete abolishment of mitochondrial function^18^.

### Metabolomics analysis

**Figure 2:**
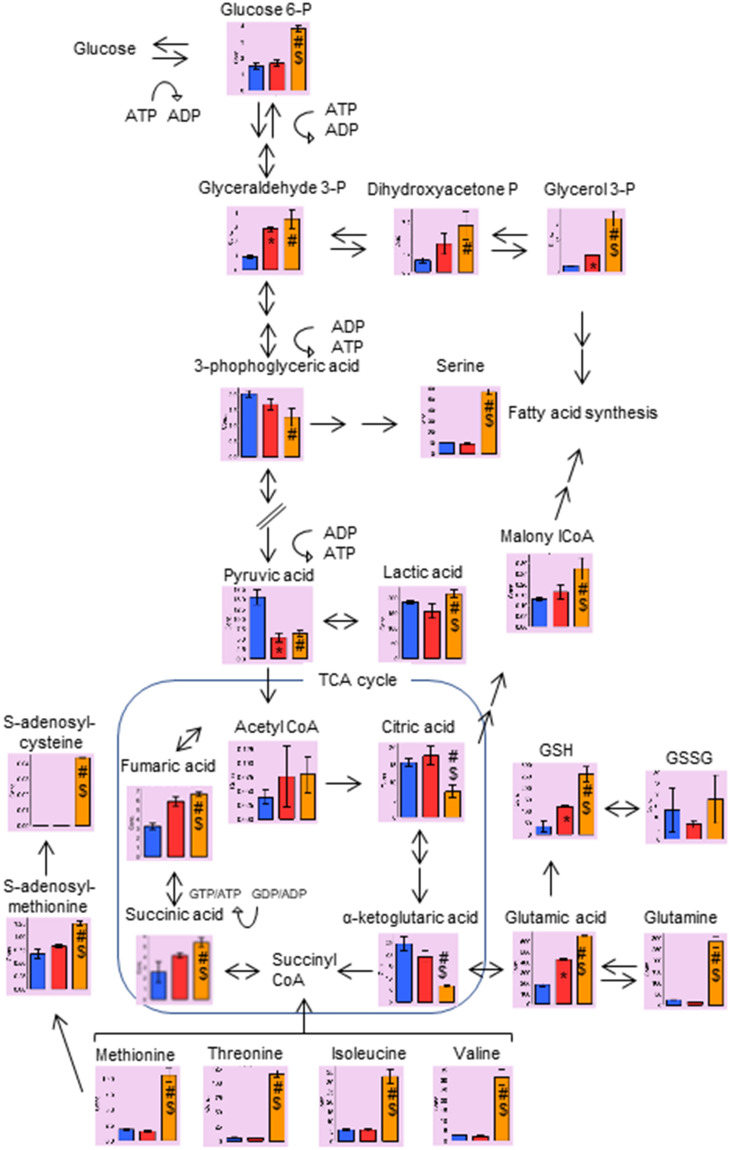
Representative data on glycolysis, the TCA cycle, lipid metabolism, glutathione synthesis and methionine metabolism. Blue, Nthy-ori 3-1; yellow, XTC.UC1; red with solid lines, TPC1; red with broken lines, TPC1 ρ0 cells. *, p < 0.01 vs. Nthy-ori 3-1; #, p < 0.01 vs. Nthy-ori 3-1; $, p < 0.01 vs. TPC1 cells. [Reproduced with permission from Human Metabolome Technologies, Inc., Tsuruoka, Japan with some modifications].

We performed C-SCOPE-based metabolomics analysis to clarify the differences in intracellular metabolites among XTC.UC1, TPC1 and Nthy-ori 3-1 cells. C-SCOPE can analyze water-soluble charged metabolites, including the glycolytic products, intermediates of the TCA cycle, as well as amino acids. All the data are illustrated in Supplementary Fig. S1 and the raw data are shown in Supplementary Table S1. The heatmap for hierarchical cluster analysis of normalized metabolic intensities, which provides a global picture of the relative amounts of metabolites, showed clear separations among the three cell lines, as did the principal component analysis (Supplementary Fig. S2A,B). Analysis of metabolite changes among the three cell lines revealed that the relative abundance of 33 metabolites was significantly (p < 0.01) altered between Nthy-ori 3-1 and TPC1 cells (19 upregulated), 45 were altered between Nthy-ori 3-1 and XTC.UC1 cells (37 upregulated) and 35 were altered between TPC1 and XTC.UC1 cells (29 upregulated). We then focused on some intriguing differences, which are summarized in Fig. 2. Regarding the glycolysis pathway, the concentration of glucose-6-phosphate was highest in XTC.UC1, indicating the highest glucose uptake. However, the concentrations of the downstream metabolites, such as glyceraldehyde 3-phosphate (G3P) and 3-phosphoglyceric acid (3PG), decreased gradually in a stepwise manner, presumably because the former was converted to dihydroxyacetone phosphate and then to glycerol 3-phosphate, and the latter to serine. G3P is thought to be used for lipid metabolism (see below), and 3PG for serine synthesis and one-carbon metabolism. Thus, substantial amounts of the metabolic intermediates in the glycolysis pathway were diverted to the production of other biomolecules in XTC.UC1 cells. Consistently, lactate was significantly (p < 0.05) but only marginally higher in XTC.UC1 than in the other two cell line. The Warburg effect was not manifest in TPC1 cells, because there were comparable glucose 6-phosphate and lactate levels between TPC1 and Nthy-ori 3-1 cells, which is consistent with a previous study^17^.

Turning to the TCA cycle, despite comparable levels of acetyl-CoA among three cell lines, citrate was lowest in XTC.UC1, indicating a block of glucose entry into the TCA cycle due to impaired OXPHOS. Citrate was likely to be transferred to the cytoplasm and converted back to acetyl-CoA and oxaloacetate, the former of which was then metabolized to malonyl CoA and, together with glycerol 3-phosphate (see above), was involved in fatty acid synthesis.

Low TCA cycle intermediates are usually replenished. For example, glutamine can be converted to glutamic acid and then to α-ketoglutaric acid (αKG), which is incorporated into the TCA cycle as an anaplerotic influx. However, in XTC.UC1 cells, high glutamine and glutamic acid but low αKG suggested little if any influx of glutamic acid into the TCA cycle. Instead, glutamic acid was converted to glutathione (GSH). Other amino acids required for GSH synthesis, cysteine and glycine, were also higher in XTC.UC1 than in TPC1 cells (see below).

Interestingly, succinic acid was higher, despite lower citrate and αKG in XTC.UC1 cells. Succinyl-CoA can be generated, as another anaplerotic flux, from branched chain amino acids (valine and isoleucine), threonine and methionine^24^, all of which were very high in XTC.UC1 cells (see below). The reaction of succinyl-CoA to succinic acid catalyzed by succinyl-CoA ligase is accompanied by ADP/GDP to ATP/GTP conversion^25^, called a mitochondrial substrate level phosphorylation (mtSLP), contributing to high-energy molecule production.

Furthermore, the reactions from methionine to S-adenosylmethionine (SAM) and then to S-adenosylhomocysteine (SAH) appeared to be accelerated in XTC.UC1 cells. SAM is a methyl donor for both DNA and histone methylation reactions that influence the epigenetic control of gene expression^26,27^. Indeed we have recently found extensive methylation of the MIEAP gene in XTC.UC1 cells^11^. In addition, together with serine (and glycine), methionine is involved in one-carbon metabolism^27^.

### Glycolysis and OXPHOS dependency

**Figure 3:**
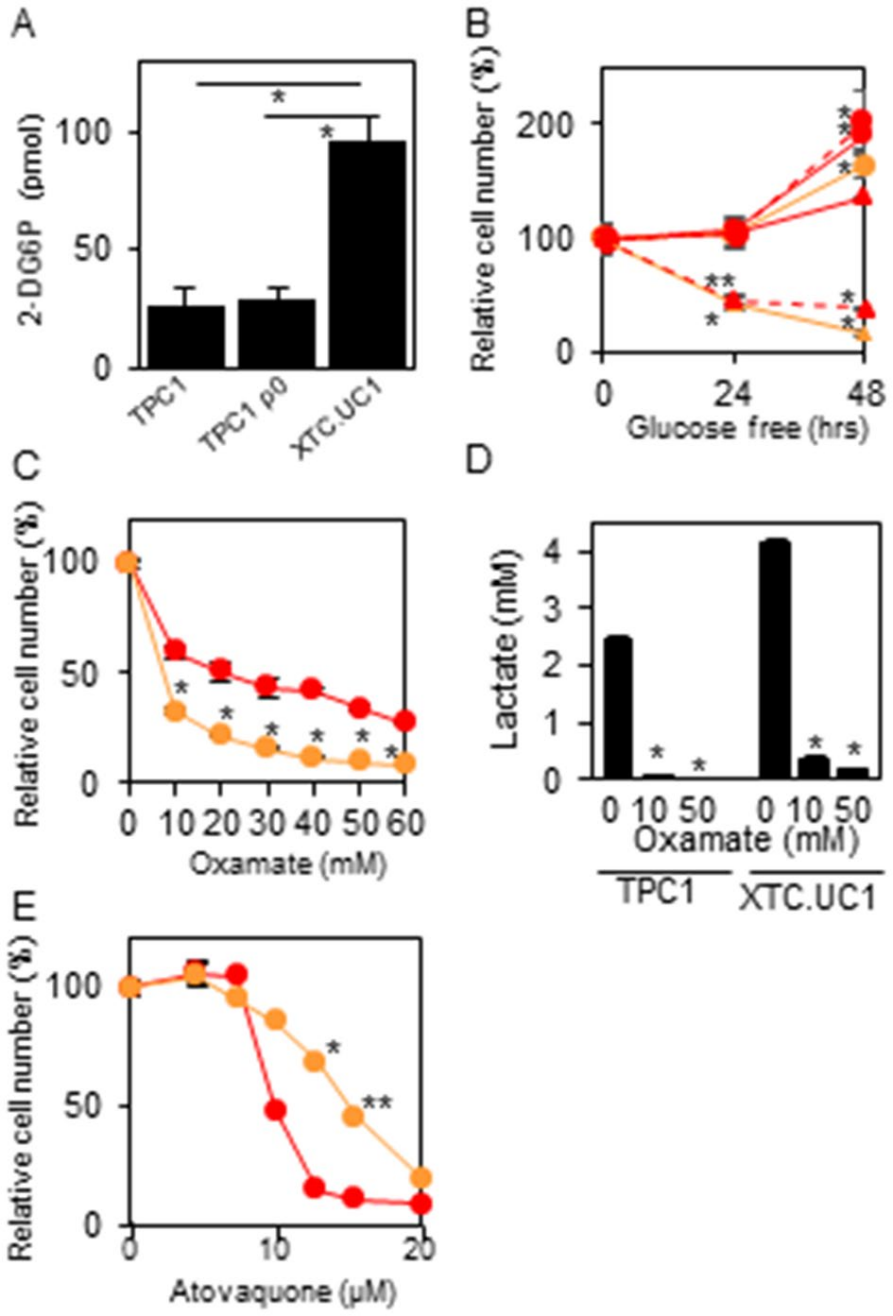
Glucose uptake (**A**), relative cell numbers in cells cultured in the presence and absence of glucose (**B**), with oxamate (**C**) or atovaquone (**E**), and the effect of oxamate on lactate production (**D**). Glucose uptake, cell number measurements and lactate measurements were performed as described in the “Materials and methods”; briefly, in (**C**–**E**), the cells were treated with 10–60 mM oxamate or 5–20 μM atovaquone for 48 h. In (**B**), red circles with solid lines, TPC1/glucose ( +); red triangles with solid lines, TPC1/glucose free; red circles with broken lines, TPC1 ρ0 cells/glucose ( +); red triangles with broken lines, TPC1 ρ0 cells/glucose free; yellow circles, XTC.UC1/glucose ( +); yellow triangles, XTC.UC1/glucose free. In (**C**,**E**): red, TPC1; yellow, XTC.UC1 cells. Data are presented as means ± SE. In (**B**): * and **, p < 0.01 and 0.05, respectively, vs. 0 h; in (**A**) and (**B**–**D**): *, p < 0.01; **, p < 0.05.

As expected from the metabolomics analysis showing the glycolytic phenotype of XTC.UC1 cells, glucose uptake was higher in XTC.UC1 cells than in TPC1 cells (Fig. 3A). In cell number measurements, cell number approximately doubled in glucose-containing medium and increased by ~ 40% in glucose-free medium in TPC1 cells in 48 h. However, in XTC.UC1 cells, although their growth pattern in glucose-containing medium was similar to that of TPC1, their cell number was reduced by ~ 40% in 48 h (Fig. 3B). Morphologically, cell death was clearly observed in XTC.UC1 cells at 24 and 48 h. To exclude the possibility that the different vulnerabilities between TPC1 and XTC.UC1 cells were due to different intrinsic phenotypes of the 2 cell types, the same experiment was performed with TPC1 ρ0 cells. Although glucose uptake was not elevated (Fig. 3A), this cell line showed a cell survival pattern similar to that of XTC.UC1 cells (Fig. 3B), clearly indicating that the difference in cell survival between TPC1 and XTC.UC1 cells shown in Fig. 3B was due to different functioning of mitochondria. Similarly, oxamate, an LDH inhibitor, killed more XTC.UC1 cells than TPC1 cells (Fig. 3C). These data are consistent with the glycolytic phenotype of XTC.UC1 cells. Indeed almost complete suppression of lactate production with oxamate was confirmed (Fig. 3D). In contrast, since TPC1 cells depend more on the TCA cycle and the ETC for energy production than XTC.UC1 cells, it was predictable that atovaquone, a complex III inhibitor of the ETC, killed more TPC1 cells than XTC.UC1 cells (Fig. 3E).

### Glutamine dependency

**Figure 4:**
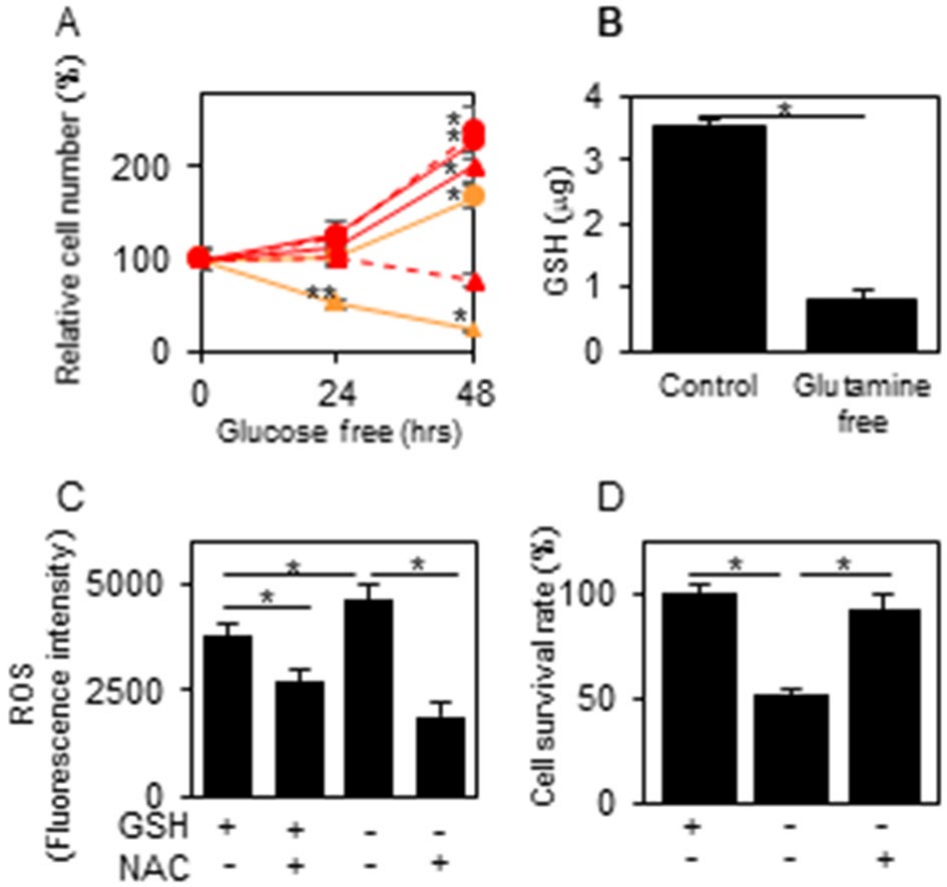
Effects of glutamine depletion on cell survival in XTC.UC1, TPC1 and TPC1 ρ0 cells (**A**), and on GSH and ROS productions and cell survival in XTC.UC1 cells treated with/without NAC (**B**–**D**). GSH and ROS measurements and cell number measurements were performed as described in the “Materials and methods”; briefly, the cells were cultured in glutamine-containing or free medium for 48 h in (**B**), and in glutamine-containing or free medium with/without 4 mM NAC for 48 h in (**C**,**D**). In (**A**): red circles with solid lines, TPC1/ glutamine (+); red triangles with solid lines, TPC1/ glutamine free; red circles with broken lines, TPC1 ρ0 cells/ glutamine (+); red triangles with broken lines, TPC1 ρ0 cells/ glutamine free; yellow circles, XTC.UC1/ glutamine (+); yellow triangles, XTC.UC1/ glutamine free. Data are presented as means ± SE. In (**A**): * and **, p < 0.01 and 0.05, respectively, vs. 0 h; in (**B**–**D**): *, p < 0.01.

The metabolomics data indicate the preferential use of glutamine for glutathione synthesis, not for the αKG supplementation, in XTC.UC1 cells. Thus, glutamine depletion showed little effect on TPC1 growth, but decreased cell number of XTC.UC1 by ~ 75% in 48 h (Fig. 4A). Again, the similar survival pattern between XTC.UC1 and TPC1 ρ0 cells indicates that the the difference in cell survival between the 2 cell lines was due to different function of mitochondria. In XTC.UC1 cells cultured in glutamine free medium, glutathione production was significantly decreased and ROS production was elevated, while cell death and ROS overproduction were cancelled by NAC, a ROS scavenger (Fig. 4B–D), demonstrating that elevated ROS was responsible for cell death by glutamine depletion.

### Amino acid metabolism

**Figure 5:**
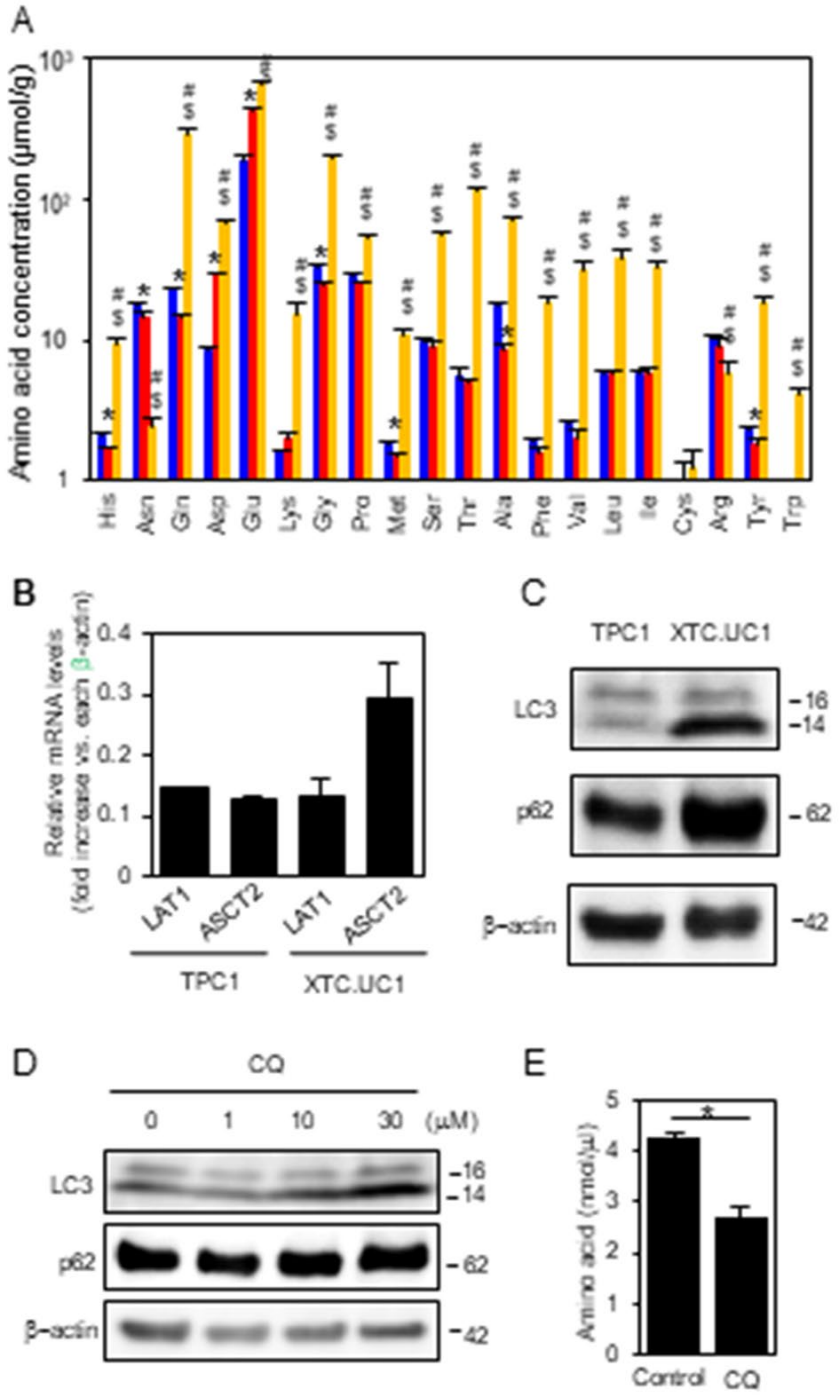
Intracellular amino acid concentrations in Nthy-ori 3-1, TPC1 and XTC.UC1 cells (**A**); mRNA expression of amino acid transporters LAT1 and ASCT2 (**B**); autophagy activities in XTC.UC1 and TPC1 cells (**B**,**C**); and the effect of the autophagy inhibitor chloroquine on autophagic activity and intracellular amino acid levels in XTC.UC1 cells (**D**,**E**). The results in (**A**) are from Supplementary Fig. S1, and RT-PCR for LAT1 and ASCT2, mRNAs, WB for LC3, p62 and β-actin and intracellular amino acid measurements were performed as described in the “Materials and methods”. In A, blue, Nthy-ori 3-1; yellow, XTC.UC1; red, TPC1 cells. Data are presented as means ± SE (n = 3 for amino acid levels) or range (n = 2 for RT-PCR). *, p < 0.01 vs. Nthy-ori 3-1; #, p < 0.01 vs. Nthy-ori 3-1; $, p < 0.01 vs. TPC1 cells. In WB in (**C**,**D**), the blots were cropped, and uncropped images are shown in Supplementary Fig. S4A,B.

As shown in Fig. 5A, most amino acid concentrations were markedly higher in XTC.UC1 cells as compared to the other two cell lines. Higher intracellular amino acid contents are likely due to higher amino acid uptake from the culture medium and/or amino acid recycling by autophagy^28^. Among the many amino acid transporters so far identified, SLC7A5 (LAT1) and SLC1A5 (ASCT2) are frequently overexpressed in cancer cells^29^. RT-PCR analysis of mRNAs for these two amino acid transporters showed higher (> twofold) expression of ASCT2 in XTC.UC1 cells than in TPC1 cells (Fig. 5B). However, since ASCT2 transports only neutral, nonpolar amino acids and not others^30^, the data shown in Fig. 5A cannot be solely explained by higher ASCT2 expression. Therefore, autophagic activity was then compared between XTC.UC1 and TPC1 cells. Higher amounts of the processed LC3-II relative to the unprocessed LC3-I indicated enhanced autophagic activity in XTC.UC1 cells as compared to the comparable levels of LC3-I and LC3-II in TPC1 cells (Fig. 5C and Supplementary Fig. S3). Indeed inhibition of autophagy with chloroquine significantly decreased amino acid levels in XTC.UC1 cells (Fig. 5D,E). It should be noted here that XTC.UC1 cells have defective mitophagy due to a loss-of-function mutation in PARK2^18^ and defective expression of MIEAP^11^. Therefore, mitochondria cannot be efficiently eliminated in this cell line, although autophagic flux is increased.

## Discussion

In the present study, we first compared the metabolic profiles among oncocytic and non-oncocytic thyroid cancer cell lines, XTC.UC1 and TPC1, and a normal thyroid cell line, Nthy-ori 3-1. Although glucose uptake was indeed higher in XTC.UC1 cells than in TPC1 cells, an enhanced glycolytic pathway not only generates the end-products of glycolysis (pyruvate, lactate and ATP) but also provides the metabolic intermediates for lipid metabolism and the serine synthetic pathway (SSP) in XTC.UC1 cells. Thus, although previous studies^15,16^ have demonstrated that XTC.UC1 cells largely rely on glycolysis for ATP production and cell survival, and our current results show that XTC.UC1 cells and TPC1 ρ0 cells are more vulnerable to glucose depletion and the LDH inhibitor oxamate than are TPC1 cells, ATP production by the glycolytic pathway may not be improved as much by higher glucose uptake. Lower ATP production by glycolysis is likely compensated by the much faster reactions of glycolysis than OXPHOS, but it is known that glycolysis intermediates are actively deviated from energy production to the provision of precursors necessary for macromolecule biosynthesis and cell proliferation in cancer cells^31^. Even in non-oncocytic thyroid cancers, the importance of lipid and serine metabolism has been reported^32–34^, and the expression levels of SSP-related enzymes have been shown to be high, especially in thyroid cancers with mutant BRAF^34^. Our data indicate that this type of metabolic reprogramming is more notable in oncocytic thyroid cancers, which is consistent with a previous article reporting the induction of the SSP by a complex I deficit^35^. It has also been reported that enzymes involved in the SSP and one-carbon metabolism are upregulated by the accumulation of OXPHOS defects in colon epithelial cells of *Lgr5-creER;Apc *^*f/f*^*; PolgA *^*mut/mut*^ mice, in which the accumulation of mtDNA mutations are accelerated^35^.

High concentrations of succinate and some amino acids that can be converted to succinyl-CoA (valine, isoleucine, threonine and methionine) imply that glutamine-independent, anaplerotic mtSLP appears to contribute to ATP production to some extent. Further studies are necessary to clarify this issue.

The lower levels of citrate and αKG in XTC.UC1 cells make sense, because impaired OXPHOS hampers the entry of glucose metabolites into the TCA cycle. Lower levels of the TCA cycle intermediates are usually replenished, and glutaminolysis is the most important anaplerotic pathway as mentioned above. αKG is used either to generate succinate and ATP/GTP from succinyl-CoA and ADP/GDP (mtSLP)^25^ and then pyruvate and lactate through malate^36^ (collectively called the oxidative TCA cycle)^28,37^, or to generate citrate for fatty acid synthesis (called reductive carboxylation)^38^, especially in the TCA cycle or OXPHOS-defective cancer cells or cancer cells treated with metformin (a complex I inhibitor)^23,39–41^. Alternatively, glutamine is used to synthesize nucleotides^42^ or glutathione^23^. Our data showing low αKG and high glutathione indicate that glutamine is preferentially used for glutathione synthesis, not for the anaplerotic pathway into the TCA cycle, in XTC.UC1 cells. This may be at least in part attributed to higher ROS production in XTC.UC1 cells^11,15^. Indeed we showed that this ROS-suppressing pathway is critical for cell survival. High glutathione synthesis has also been demonstrated in renal oncocytoma with high intracellular ROS levels^20,43,44^. The higher concentration of fumarate observed in XTC.UC1 cells (Fig. 2) has also been reported to participate in redox homeostasis by activating glutathione peroxidase 1, which eliminates ROS with glutathione^45^.

Higher concentrations of intracellular amino acids have been reported in cancer cells in general^46^, and glycolytic intermediates supply cancer cells with the amino acids required for fast cellular proliferation^47^. We revealed here that both higher expression of the amino acid transporter ASCT2 and augmented autophagic activity contributed to higher amounts of amino acids in XTC.UC1 cells, although overexpression of LAT1 and ASCT2 have previously been reported even in non-oncocytic thyroid cancer cells and are correlated with poor patient prognosis^48–51^. A previous study has revealed higher autophagic activity in XTC.UC1 than in Nthy-ori 3-1 cells, which compensates dysfunctional mitophagy by the PARK2 mutation^18^. As mentioned above, these amino acids are likely used for glutathione synthesis, TCA cycle anaplerosis, methylation, etc*.,* in XTC.UC1 cells.

While we were performing this research project, Addie et al.^23^ published a metabolomic analysis of thyroid cancer cell lines with or without “near-homozygous genome” including XTC.UC1 cells (lower complex I and II activities), FTC-236 cells (lower complex I, II and IV activities), and SW579 and BHP2-7 cells (both having normal OXPHOS). A near-homozygous genome is a chromosomal abnormality frequently observed in oncocytoma^3,52^, and BHP2-7 cells are genetically identical to TPC1 cells^53^. Although it is very difficult to interpret their data because each cell line exhibited unique metabolic characteristics. When compared the data on XTC.UC1 and TPC1 (BHP2-7) cells between our study and theirs, there are some inconsistent data between 2 studies. For examples, although the concentrations of many amino acids were commonly increased in XTC.UC1 compared to TPC1 (BHP2-7) cells in both studies, those of asparagine and arginine were declined in ours, and glutamine, serine and tryptophan were reduced in theirs. Furthermore, lactate was higher in ours but lower in theirs, and citrate was lower in ours but higher in theirs in XTC.UC1 compared to TPC1 (BHP2-7) cells (citrate; see below).

The reasons for these discrepancies are unknown; they may represent heterogeneity within these cell lines among different laboratories. Some characteristics of XTC.UC1 and/or TPC1 cells might have changed during a long period of in vitro cell culture. Indeed there is one difference in genotyping data of XTC.UC1 cells between Cellosaurus and ours (Supplementary Fig. S2).

In conclusion, our data demonstrate that XTC.UC1 cells exhibit higher glycolytic activity than TPC1 cells, but the glycolytic intermediates are utilized not only for the completion of glycolysis but also for supplementation of branching pathways such as lipid metabolism and the SSP. Growth, survival and redox homeostasis of XTC.UC1 cells depend more on both glucose and glutamine than in TPC1 cells. Finally, higher intracellular amino acid contents, which are due to higher glycolytic activity, and enhanced cellular uptake and autophagy, provide the building blocks for macromolecule and energy production. These metabolic alterations are needed for oncocytic cancer cells to compensate for their defective mitochondrial function and to alleviate excess oxidative stress.

## Materials and methods

### Cell lines used in this study

The oncocytic thyroid cancer cell line XTC.UC1 (kindly provided by prof. Porcelli AM, Bologna University, Italy)^54^, the non-oncocytic papillary thyroid cancer cell line TPC1 (kindly provided by prof. Fagin JA, Sloan Kettering Cancer Center, NY, USA)^55^ and the immortalized normal thyroid cell line Nthy-ori 3-1 (from the Health Protection Agency Culture Collections, Salisbury, UK)^56^ were used in this study. TPC1 cells have the *RET-PTC1* chromosome translocation. XTC.UC1 cells have mutations in the *ND1*, *cytochrome b*^15^ and *p53* (a homozygous 451C > A, corresponding to Pro151Thr) genes^57^ but no point mutations in *BRAF* or *RAS*, and no chromosomal translocation in *RET*, *NTRK1* or *ALK* (data not shown). The authenticity of TPC1 was confirmed by short tandem repeat profiling previously^58^, and those of XTC.UC1 and Nthy-ori 3-1 were examined in this study and found to be the same as those in the Cellosaurus except for one focus in XTC.UC1 (FGA) (Supplementary Table S2) (National Institute of Biomedicine Innovation, Ibaraki, Japan).

XTC.UC1 cells were cultured in DMEM (08459-64, Nacalai Tesque Inc., Kyoto, Japan) with 10% FBS and antibiotics, TPC1 cells in RPMI 1640 (30264-85, Nacalai Tesque) with 5% FBS and antibiotics and Nthy-ori 3-1 cells in RPMI 1640 with 10% FBS and antibiotics. For glucose-free culture, RPMI 1640 (No Glucose) with L-glutamine (09892-15, Nacalai Tesque) and DMEM (No Glucose) with L-glutamine (09891-25, Nacalai Tesque) were used, and for glutamine-free culture, RPMI 1640 without L-Gln (05176-25, Nacalai Tesque) and DMEM (4.5 g/L glucose) without L-Gln (08488-55, Nacalai Tesque) were used. None of media contained pyruvate. Cell culture was performed at 37 °C in a humidified incubator with 5% CO_2_.

### Metabolite measurements

Metabolic extracts were prepared from ~ 5 × 10^6^ cells with methanol containing Internal Standard Solution (H3304–1002, Human Metabolome Technologies (HMT), Inc., Tsuruoka, Japan), transferred into a microfuge tube and centrifuged at 2300×*g* and 4 °C for 5 min. The upper aqueous layer was then centrifugally filtered through a Millipore 5-kDa cutoff filter at 9100×*g* at 4 °C for 120 min to remove proteins. The filtrate was centrifugally concentrated and resuspended in 50 μL dH_2_O, and analyzed using a capillary electrophoresis (CE)-connected ESI-TOFMS and CE-MS/MS system (HMT, CARCINOSCOPE). Cationic compounds were analyzed in the positive mode of CETOFMS and anionic compounds were analyzed in the positive and negative modes of CE-MS/MS. The data were analyzed by MasterHands ver.2.17.1.11 (Keio University, Tsuruoka, Japan) and MassHunter Quantitative Analysis B.06.00 (Agilent Technologies, Santa Clara, CA), and the peak area of each metabolite was calculated. The peak area was then normalized to an internal standard, and the metabolite concentrations were calculated using standard curves. Overall, 116 compounds were measured^59,60^.

### Generation of TPC1 ρ0 cells

TPC1 cells in a 10 cm dish (1 × 10^6^ cells) were cultured for 8 weeks with medium containing 0.5 μg/mL ethidium bromide (315-90051, Nippon Gene, Tokyo, Japan), 50 μg/mL uridine (U3003, Sigma-Aldrich, St Louis, MD, USA) and 1 mM sodium pyruvate (P8574, Sigma-Aldrich). Medium was changed twice a week. After 8 weeks, almost complete elimination of mtDNA was confirmed with PicoGreen staining (P7581, Thermo Fisher, MA, USA)^61^ and real time PCR^62^. Supplementary Fig. S4 shows the absence of mtDNA (A) in PicoGreen staining and an extremely lower amount of mtDNA detected by PCR (B) in ethidium bromide-treated cells. The cells were then cultured for another 2 weeks without ethidium bromide, and the absence of mtDNA was again confirmed with PicoGreen staining and PCR. Thereafter these TPC1 ρ0 cells were kept in RPMI 1640 supplemented with 5% FBS, antibiotics 50 μg/mL uridine and 1 mM sodium pyruvate.

To quantify mtDNA by PCR, total DNA was isolated from cells using a DNeasy Blood & Tissue Kit (69504, Qiagen, Valencia, CA) according to the manufacturer’s instructions, and then used for PCR amplification of *12S ribosomal DNA* (*12S rDNA*) encoded by mtDNA and and *β-ACTIN* encoded by nuclear DNA (nDNA) with TB-Green Premix Ex Taq II (RR820A, Takara Bio, Kusatsu, Japan) and a Thermal Cycler Dice Real-Time System (Takara Bio). Then mtDNA/nDNA ratio was used to estimate the relative mtDNA copy number, *i.e.*, the relative amounts of mitochondria. The primers used were described previously^11^; *12Sr* DNA primers are 5ʹ-TAACCCAAGTCAATAGAAGCC-3ʹ (forward) and 5ʹ-CTAGAGGGATATGAAGCACC-3ʹ (reverse), and human *β-ACTIN* primers are 5ʹ-GAGCGGGAAATCGTGCGTGAC-3ʹ (forward) and 5ʹ-GGAAGGAAGGCTGGAAGAGTG-3ʹ (reverse). The PCR conditions were 40 cycles of denaturation at 95 °C for 30 s, annealing at 55 °C for 30 s and extension at 72 °C for 30 s. The correct sizes of PCR products were also confirmed with 2% agarose gel electrophoresis (data not shown).

### Mitochondrial stress test

The mitochondrial oxygen consumption rate (OCR) was measured using a Seahorse XF-96 extracellular flux analyzer (Seahorse Bioscience, North Billerica, MA, USA). The cells were seeded in an XF-96 plate (0.8 × 10^4^ cells/well) and incubated in regular culture medium with 5% FBS for 24 h. Meanwhile, the sensor cartridge was calibrated with calibration buffer supplied by the manufacturer at 37 °C without CO_2_. For measurement of OCR, the culture medium was replaced with the XF Base Medium (Seahorse Bioscience) containing 1 mM sodium pyruvate, 10 mM glucose and 2 mM glutamine (all from Seahorse Biosciences). After 1 h culture without CO_2_, readings were taken at the base line and after the addition of various chemicals such as 2 μM oligomycin, 0.25 μM phenylhydrazone or 0.5 μM rotenone/antimycin (all from Seahorse Bioscience). OCR was automatically calculated and recorded using the sensor cartridge and Seahorse XF-96 software. The plates were saved, and the cell number was determined with a Cell Counting Kit-8 (Dojindo, Kumamoto, Japan) according to the manufacturer’s protocol.

### Cell number measurements

The cells in a 96 well plate (3 × 10^3^ cells/well) were treated with atovaquone (5–20 μM; A2545, Tokyo Chemical Industry Co., Ltd., Tokyo, Japan)) or oxamate (10–60 mM; O2751, Sigma-Aldrich) or cultured in glucose- or glutamine-free medium for up to 48 h. The viable cell numbers were then determined with the water-soluble tetrazolium salt (WST) assay using a Cell Counting Kit-8. The plates were read with a 2030 Multilabel Plate Reader ARVO X3 (Perkin Elmer, Branchburg, NJ) at 450 nm.

### Glucose uptake measurements

The cells in a 96 well plate (2.5 × 10^2^ TPC1 and 2 × 10^3^ XTC.UC1 cells/well) were incubated in regular culture medium. Five days later, the cell numbers for each cell line was confirmed to be approximately the same. The cells were washed twice with PBS containing Ca^+^ and Mg^+^ (PBS(+)), preincubated with KRPH buffer containing 2% BSA for 40 min, and then cultured with 1 mM 2-deoxyglucose for 20 min. After lysing the cells, intracellular glucose contents were measured with a Glucose Uptake Colorimetric Assay Kit (K676-100, BioVision, Inc., Waltham, MA) according to the manufacturer’s protocol. The plates were read with a 2030 Multilabel Plate Reader ARVO X3 (PerkinElmer) at 412 nm.

### Lactate measurements

The cells in a 96 well plate (5 × 10^3^ cells/well) were incubated with or without 10 and 50 mM oxamate for 48 h. The culture media were then collected and diluted with H_2_O accordingly. Lactate concentrations in the media were measured using by a Lactate Assay Kit-WST (L256, Dojindo) according to the manufacturer's instruction. The plate was read with a 2030 Multilabel Plate Reader ARVO X3 (PerkinElmer) at 450 nm.

### GSH measurements

The cells in a 96 well plate (2.5 × 10^3^ cells/well) were incubated in regular culture medium or glutamine-free medium with or without 4 mM N-acetyl cysteine (NAC; A7250, Sigma–Aldrich) for 48 h. GSH levels were then measured with a GSH-Glo™ Glutathione Assay Kit (V6911, Promega, Madison, WI) according to the manufacturer's protocol. The plates were read with a 2030 Multilabel Plate Reader ARVO X3 (PerkinElmer) to detect luminescence.

### ROS measurements

The cells in a 96-well plate (5 × 10^4^ cells/well) were washed with PBS ( +), incubated with 5 μM 2’,7’-dichlorofluorescin diacetate (C6827, Thermo Fisher Scientific, Waltham, MA) in PBS ( +) for 0.5 h at 37 °C and washed again with PBS ( +). The plates were read with a 2030 Multilabel Plate Reader ARVO X3 (PerkinElmer) at excitation and emission wavelengths of 485 and 528 nm, respectively. The results were expressed as relative values vs. the control.

### Intracellular L-amino acid measurements

XTC.UC1 cells in a 10 cm dish were cultured with or without 1–30 μM chloroquine (08660-04, Nacalai Tesque) for 48 h. Then 2 × 10^6^ cells were suspended in 500 μL of L-amino acid assay buffer (K639-100-1, Bio Vision Inc.), completely lysed with a homogenizer and centrifuged. The supernatants obtained were used for amino acid quantitation using an L-Amino Acid Quantitation Kit (BioVision Inc.) according to the manufacturer's instructions. The plates were read with a 2030 Multilabel Plate Reader ARVO X3 (PerkinElmer) at excitation and emission wavelengths of 535 and 587 nm, respectively.

### Western blotting

Expression of LC3, p62 and β-actin were determined by immunoblotting with 40 μg cell lysates as described previously^63^. After sodium dodecyl sulfate polyacrylamide gel electrophoresis and transfer onto a polyvinylidene fluoride membrane (Bio-Rad Laboratories Inc., Tokyo, Japan), the membranes were blotted with the following antibodies and chemiluminescence system (Thermo Fisher Scientific, Rockford, IL). The primary and secondary antibodies used were (i) polyclonal rabbit anti-LC3 (PM036, MBL International, Woburn, MA; 1:1000) and polyclonal goat anti-rabbit horseradish peroxidase (HRP)-conjugated IgG (7074, Cell Signaling Technology, Danvers, MA; 1:1000) for LC3; (ii) polyclonal guinea pig anti-p62 (GP62-C, Progen, Heidelberg, Germany; 1:1000) and polyclonal rabbit anti-guinea pig HRP-conjugated IgG (61-4620, Innovative Research, Novi, MI; 1:1000) for p62; and (iii) monoclonal mouse anti β-actin (C4, Santa Cruz Biotechnology, Dallas, TX; 1:1000) and polyclonal horse anti-mouse HRP-conjugated IgG (7076, Cell Signaling Technology; 1:1000) for β-actin. Protein detection was performed using LAS-3000 (FUJIFILM) and quantified using NIH ImageJ software. A molecular weight Novex® Sharp pre-stained Protein Standard (LC5800, Thermo Fisher Scientific) was run in parallel.

### Quantitative real-time RT-PCR

Total RNA was extracted from cells using ISOGEN reagent (311-02501, Nippon Gene, Tokyo, Japan). cDNA was synthesized from 500 ng total RNA with SuperScript III reverse transcriptase (18080-400, Thermo Fisher Scientific) using random hexamers. Quantitative PCR was then carried out using a Thermal Cycler Dice Real-Time System (Takara Bio) using TB Green Premix EX Taq II (RR820A, Takara Bio). The primers used were 5′-ACCATGGTTCTGGTCTCCTG-3′ and 5′-GGGCAAAGAGTAAACCCACA-3′ for ASCT2, and 5′-GGCCTTCATCGCAGTACATC-3′ and 5′-GCCTTCACGCTGTAGCAGTT-3′ for LAT1. The PCR conditions were 40 cycles of denaturation at 95 °C for 15 s, annealing at 55 °C for 15 s, and extension at 72 °C for 20 s. The cycle threshold values, which were determined using second derivative, were used to calculate the normalized expression of the indicated mRNAs using Q-Gene soft-ware using *β-actin* for normalization. The negative control reaction was done without reverse transcription in parallel. The correct sizes of PCR products were confirmed with 2% agarose gel electrophoresis (data not shown).

### Statistical analysis

All data are expressed as means ± SE (n = 3) or ranges (n = 2) and differences between groups were examined for statistical significance using Scheffe's multiple comparison test. A p-value < 0.05 was considered statistically significant. All the experiments were repeated at least twice with essentially the same results.

## Supplementary Information

Supplementary Information 1.

Supplementary Information 2.

## Data Availability

All data generated or analyzed during this study are included in this published article.
