# Supplementary material for: Characterization of metabolic reprogramming by metabolomics in the oncocytic thyroid cancer cell line XTC.UC1
Source: Sci Rep. 2023 Jan 4;13:149. doi: 10.1038/s41598-023-27461-2 (PMC9813134; doi:10.1038/s41598-023-27461-2)
Supplement: Supplementary file 1 — Supplementary Information 1. [file 41598_2023_27461_MOESM1_ESM.pdf]

Supplementary Data for

Characterization of Metabolic Reprogramming by Metabolomics in an Oncocytic Thyroid Cancer Cell Line XTC.UC1

Tomomi Kurashige, Mika Shimamura, Koichiro Hamada, Michiko Matsuse, Norisato Mitsutake, Yuji Nagayama

This PDF file includes:

Supplementary Figure 1. The overall energy metabolism chart.

Supplementary Figure 2. The heatmap and the principle component analysis of the metabolome data.

Supplementary Figure 3. Uncropped images of Fig. 5C and D.

Supplementary Figure 4. PicoGreen staining and real time PCR to detect nDNA and mtDNA in control TPC1 and those treated with ethidium bromide (TPC1 ρ0 cells).

Supplementary Table 2. The short tandem repeat profiling of 3 cell lines.

Other Supplementary data presented in separate files:

Supplementary Table 1. Absolute values for metabolic substances.

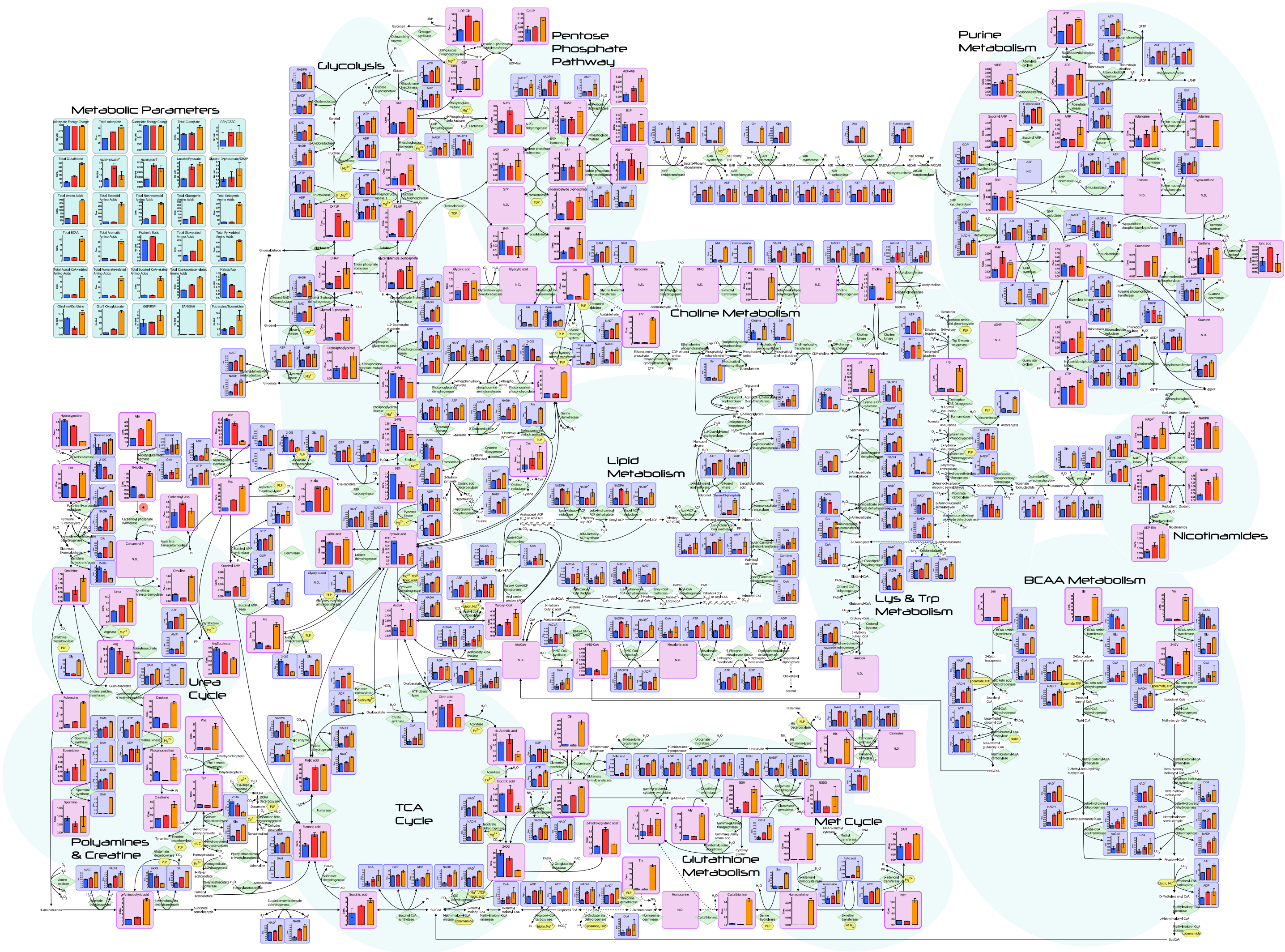

Supplementary Fig. 1. Overall energy metabolism chart. Blue, yellow and red indicate Nthy-ori 3-1, XTC.UC1 and TPC1 cells, respectively. [Reproduced with permission from Human Metabolome Technologies, Inc., Tsuruoka, Japan].

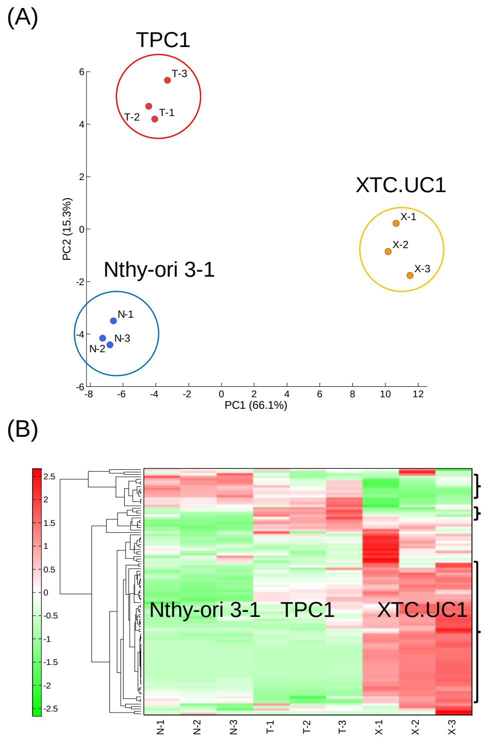

Supplementary Fig. 2. Heatmap (A) and the principal component analysis (B) of the metabolome data. Blue, yellow and red indicate Nthy-ori 3-1, XTC.UC1 and TPC1 cells, respectively.

A

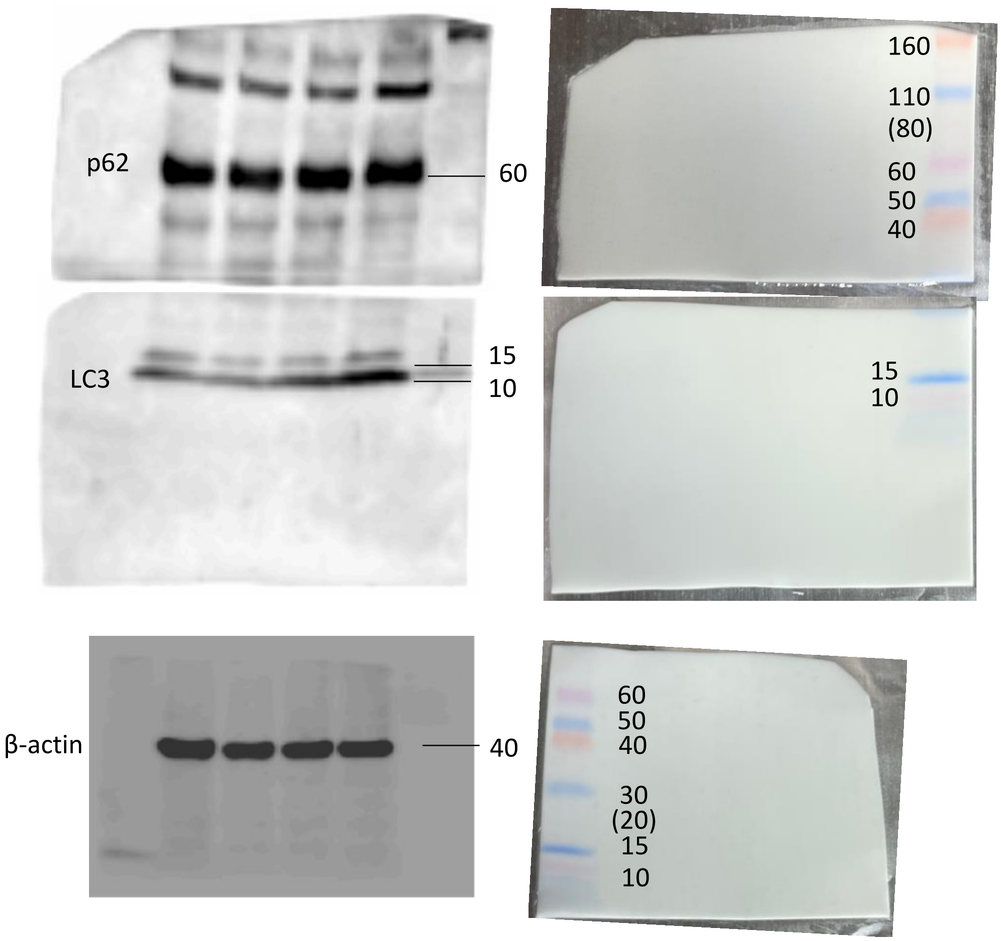

B

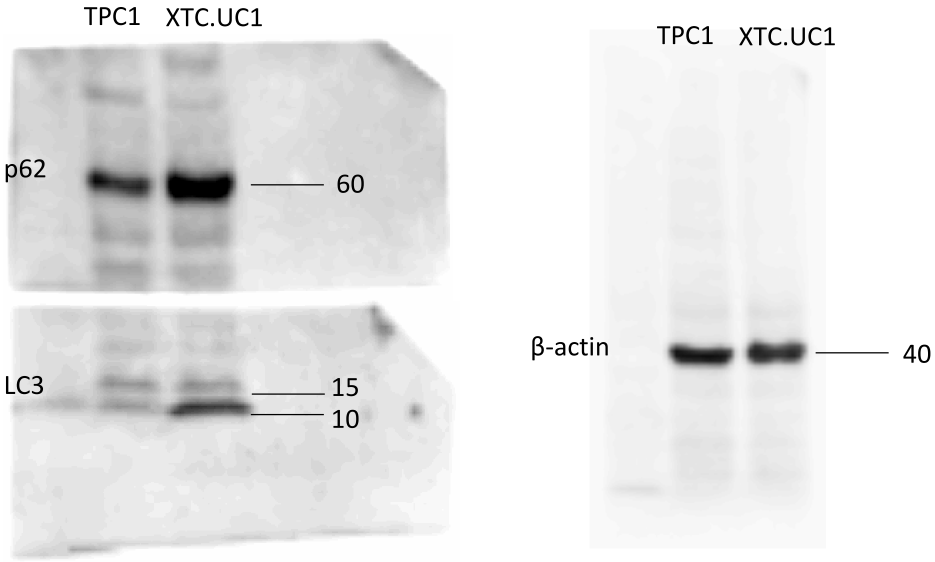

Supplementary Fig. 3. Uncropped images of Fig. 5C (A) and D (B). In each panel, 2 sets of samples were run in parallel in a gel, and after transfer the membranes were cut into 2 pieces; one set was used to detect β-actin, and the other was further cut into 2 pieces and used to detect p62 and LC3. The molecular markers are also shown in (A).

A

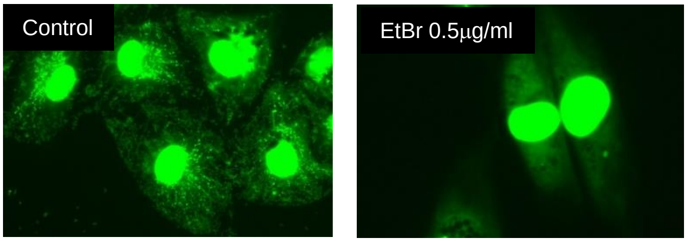

B

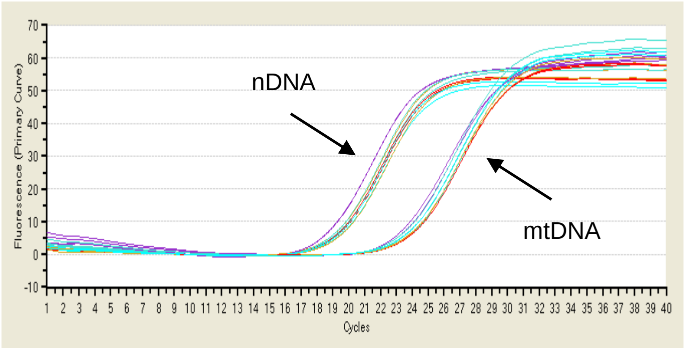

Supplementary Fig. 4. PicoGreen staining and real time PCR to detect nDNA and mtDNA in control TPC1 cells and in those treated with ethidium bromide (TPC1 $\rho 0$ cells). (A) PicoGreen staining shows brightly stained nuclei surrounded by numerous bright punctate cytoplasmic spots indicating the presence of mtDNA in control cells (left), but their absence in ethidium bromide-treated cells (right). (B) Real time PCR for nDNA and mtDNA in 5 clones of ethidium bromide-treated cells. The copy number of mtDNA was calculated as $2 \times 1/2^{(\text{average CT value for mtDNA – that for nDNA})} = 2 \times 1/2^{23.81-19.01} = 0.07$, showing the efficient elimination of mtDNA. One of these 5 clones was used in subsequent experiments.

Supplementary Table 2. The short tandem repeat profiling of 3 cell lines.

	TPC1		XTC.UC1		Nthy-ori 3-1	
	Cellosaurus data *	Our data	Cellosaurus data	Our data	Cellosaurus data	Our data
CSF1PO	11, 12	11, 12	11	11	12	12
D3S1358	16, 17	16, 17	15	15	14, 16	14, 16
D5S818	8, 10	8, 10	11, 12	11, 12	11	11
D7S820	11	11	11	11	7, 12	7, 12
D8S1179	11, 17	11, 17	12	12	12	12
D13S317	11, 12	11, 12	12	12	11	11
D18S51	13, 16	13, 16	13, 17	13, 17	12, 13	12, 13
FGA	20, 21	20, 21	24.2 **	25 **	14	14
TH01	9	9	9.3	9.3	21, 22	21, 22
TPOX	11	11	8	8	7 (ECACC; PubMed=21868764) 7,9.3 (PubMed=3073244)	7,9.3
vWA	14, 18	14, 18	18	18	9	9
CSF1PO	11, 12	11, 12	11	11	16, 18	16, 18

*, obtained from https://www.cellosaurus.org/. **, a difference between the Cellosaurus and our data.
